# Interpreting infection–disease associations in psoriasis and atopic dermatitis

**DOI:** 10.1016/j.jdin.2026.05.029

**Published:** 2026-06-25

**Authors:** Rahib K. Islam, Kazi N. Islam, Nicholas J. Culotta, Christopher J. Haas, Jashin J. Wu

**Affiliations:** a School of Medicine, Louisiana State University Health Sciences Center, New Orleans, Louisiana; b Department of Dermatology, Louisiana State University Health Sciences Center, New Orleans, Louisiana; c Agricultural Research Development Program, Central State University, Wilberforce, Ohio; d California Dermatology, Corona, California; e Department of Dermatology, University of Miami Miller School of Medicine, Miami, Florida

**Keywords:** atopic dermatitis, dermatology, infection risk, inflammatory skin disease, Janus kinase inhibitor, psoriasis, streptococcal pharyngitis

*To the Editor:* We read with great interest the research letter by Zhou et al, who synthesized 29 cohort studies and reported that infection was associated with increased risk of psoriasis (hazard ratio [HR] 1.64, 95% confidence interval [CI] 1.32-2.04, *P* < .001) and atopic dermatitis (AD; HR 1.23, 95% CI 1.17-1.31, *P* < .001).^1^ Their findings are clinically important because they encourage dermatologists to consider infectious exposures as more than incidental historical details. We commend the authors for highlighting this emerging area and suggest that future studies can build on their work by more explicitly separating infection as an exposure, trigger, complication, and marker of health care contact.

Temporality is central. Established inflammatory skin disease can itself increase infection risk, creating potential reverse causation when infection is measured shortly before first coded diagnosis. In population-based cohorts, psoriasis and hospital-managed AD have each been associated with higher risks of serious or systemic infections.^2^^,^^3^ Therefore, infections occurring near the time of diagnosis may reflect prodromal disease activity, barrier compromise, comorbidity, treatment exposure, or increased medical surveillance rather than a purely upstream causal exposure.

Phenotype and infection type also matter. In psoriasis, streptococcal pharyngitis is most biologically aligned with guttate psoriasis, whereas pooled “psoriasis” outcomes may include chronic plaque disease, pustular disease, inverse disease, and psoriatic arthritis. In AD, microbial exposure is closely linked to epidermal barrier dysfunction and colonization: a systematic review and meta-analysis found markedly increased odds of *Staphylococcus aureus* carriage in lesional AD skin compared with healthy controls.^4^ Combining bacterial, viral, fungal, and parasitic infections without sufficient attention to anatomic site, severity, timing, and diagnostic certainty may therefore obscure disease-specific mechanisms.

Treatment exposure is an additional interpretive layer. The BioDay registry demonstrated higher infection rates among patients with moderate-to-severe AD treated with Janus kinase inhibitors compared with dupilumab, illustrating that targeted therapies can materially reshape infection risk.^5^ Future observational studies and meta-analyses should routinely report topical and systemic corticosteroid use, conventional immunosuppressants, biologic class, Janus kinase inhibitor exposure, vaccination status, antimicrobial use, disease severity, phenotype, time from infection to diagnosis, and health care utilization.

Zhou et al have provided a useful synthesis and an important platform for future work. Standardized reporting of infection subtype, disease phenotype, treatment exposure, and temporality would make subsequent pooled estimates more clinically interpretable and help distinguish causal infection-triggered disease from reverse causation or ascertainment bias.

## Conflicts of interest

None disclosed.
